# Performance of Copeptin for Early Diagnosis of Acute Coronary Syndromes: A Systematic Review and Meta-Analysis of 14,139 Patients

**DOI:** 10.3390/jcdd9010006

**Published:** 2021-12-27

**Authors:** Lukasz Szarpak, Marcin Lapinski, Aleksandra Gasecka, Michal Pruc, Wiktoria L. Drela, Mariusz Koda, Andrea Denegri, Frank W. Peacock, Miłosz J. Jaguszewski, Krzysztof J. Filipiak

**Affiliations:** 1 Institute of Outcomes Research, Maria Sklodowska-Curie Medical Academy, 03-411 Warsaw, Poland; 2 Research Unit, Maria Sklodowska-Curie Bialystok Oncology Center, 15-027 Bialystok, Poland; kodamar2@gmail.com; 3 Research Unit, Polish Society of Disaster Medicine, 05-806 Warsaw, Poland; m.pruc@ptmk.org; 4 1st Chair and Department of Cardiology, Medical University of Warsaw, 02-097 Warsaw, Poland; s071447@student.wum.edu.pl (M.L.), gaseckaa@gmail.com (A.G.); 5 Laboratory of Experimental Clinical Chemistry, Amsterdam University Medical Center, 1105 Amsterdam, The Netherlands; 6 Students Research Club, Maria Sklodowska-Curie Medical Academy, 04-311 Warsaw, Poland; drelawiktorialaura@gmail.com; 7 Cardiology Division, Department of Biomedical, Metabolic and Neural Sciences, University of Modena and Reggio Emilia, Policlinico di Modena, 41121 Modena, Italy; denegri.andrea@aou.mo.it; 8 Henry JN Taub Department of Emergency Medicine, Baylor College of Medicine, Houston, TX 77030, USA; frankpeacock@gmail.com; 9 1st Department of Cardiology, Medical University of Gdansk, 80-211 Gdansk, Poland; jamilosz@gmail.com; 10 Institute of Clinical Medicine, Maria Sklodowska-Curie Medical Academy, 00-001 Warsaw, Poland; krzysztof.filipiak@uczelniamedyczna.com.pl

**Keywords:** copeptin, biomarker, acute coronary syndrome, acute myocardial infarction, systematic review

## Abstract

Diagnosis of acute coronary syndrome (ACS) based on copeptin level may enable one to confirm or rule-out acute myocardial infarction (AMI) with higher sensitivity and specificity, which may in turn further reduce mortality rate and decrease the economic costs of ACS treatment. We conducted a systematic review and meta-analysis to investigate the relationship between copeptin levels and type of ACS. We searched Scopus, PubMed, Web of Science, Embase, and Cochrane to locate all articles published up to 10 October 2021. We evaluated a meta-analysis with random-effects models to evaluate differences in copeptin levels. A total of 14,139 patients (4565 with ACS) were included from twenty-seven studies. Copeptin levels in AMI and non-AMI groups varied and amounted to 68.7 ± 74.7 versus 14.8 ± 19.9 pmol/L (SMD = 2.63; 95% CI: 2.02 to 3.24; *p* < 0.001). Copeptin levels in the AMI group was higher than in the unstable angina (UAP) group, at 51.9 ± 52.5 versus 12.8 ± 19.7 pmol/L (SMD = 1.53; 95% CI: 0.86 to 2.20; *p* < 0.001). Copeptin levels in ST-elevation myocardial infarction (STEMI) versus non-ST elevation myocardial infarction (NSTEMI) patient groups were 54.8 ± 53.0 versus 28.7 ± 46.8 pmol/L, respectively (SMD = 1.69; 95% CI: = 0.70 to 4.09; *p* = 0.17). In summary, elevated copeptin levels were observed in patients with ACS compared with patients without ACS. Given its clinical value, copeptin levels may be included in the assessment of patients with ACS as well as for the initial differentiation of ACS.

## 1. Introduction

Acute coronary syndromes (ACS) represent the leading cause of morbidity and mortality worldwide [1] and although the relative incidences of ST-elevation myocardial infarction (STEMI) and non-ST elevation myocardial infarction (NSTEMI) are decreasing and increasing, respectively [2,3], they are still responsible for almost 1.8 million annual deaths [4]. Instant diagnostics, rapid and accurate assessment of electrocardiographic (ECG) parameters, and troponin are crucial in the identification of patients suffering from acute myocardial infarction (AMI) [3,4]. Distinguishing those requiring immediate intervention and hospitalization is a vital issue both for patients and clinicians [5].

According to current guidelines, besides ECG, cardiac biomarkers and in particular troponins are also used to diagnose AMI [6,7]. However, ruling out AMI with ECG and troponins is time-consuming owing to the need for serial blood sampling to determine the changes in troponin concentrations, especially in patients with non-ST-elevation ACS. The release of high-sensitivity cardiac troponin T (hs-cTnT) is delayed in comparison with myocardial necrosis onset [8]. Troponin is very sensitive, which leads to a high number of false-positive results [9,10]. Therefore, a more reliable indicator of AMI is needed to identify patients who require treatment at an early stage.

Recent studies reported that the level of copeptin, the C-terminal part of arginine-vasopressin (AVP), may be elevated within 30 min after the onset of chest pain in patients with AMI [11,12] as a result of endogenous stress response [13]. Copeptin does not require serial sampling, in contrast to troponin [14] and may represent an accurate anchor point to diagnose AMI in patients admitted to ED. Hence, diagnosis of AMI based on copeptin level may enable to confirm or rule-out ACS with higher sensitivity and specificity, which may in turn further reduce mortality rate and decrease the economic costs of AMI treatment. The aim of our meta-analysis was to evaluate the diagnostic value of copeptin in ACS.

## 2. Materials and Methods

This systematic review and meta-analysis follows the recommendation of the Preferred Reporting Items for Systematic Review and Meta-Analyses (PRISMA) statement [15]. Before commencing the study, all authors agreed on the analysis methods and the inclusion and exclusion criteria to be used.

### 2.1. Search Strategy

We search evidence up to 10 October 2021 in the following databases: Scopus, PubMed, Web of Science, Embase, and Cochrane. The search was conducted using the following terms: “copeptin” OR “copeptins” OR “Glycopeptides” OR “Glycopeptide” OR “C-terminal provasopressin” AND “acute myocardial infarction” OR “AMI” OR “myocardial infarction” OR “MI” OR “STEMI” OR “ST-Elevation” OR “non-ST segment elevation” OR “NSTEMI” OR “ACS” OR “acute coronary syndrome”. All references were saved in an EndNote (End Note, Inc, Philadelphia, PA) library used to identify the duplicates. We did not limit the search by language or publication date. We also manually searched the reference list of identified trials for other potentially eligible studies.

### 2.2. Inclusion Criteria

Studies included in this meta-analysis met the following PICOS criteria: (1) PARTICIPANTS, patients > 18 years of age; (2) INTERVENTION, patients with AMI; (3) COMPARISON, patients without AMI; (4) OUTCOMES, detailed information for copeptin levels; and (5) STUDY DESIGN, randomized controlled trials or observational studies comparing copeptin levels in patients with and without AMI or comparing copeptin levels in patients with different AMI groups.

This review excluded the following types of studies: (1) papers not containing a comparator group; (2) conference or poster papers; (3) reviews; (4) case reports; and (5) articles not containing original data.

### 2.3. Data Extraction

Two authors (L.S. and S.B.) independently reviewed the selected trials and extracted the data of interest. The extraction of data was performed using a pre-piloted Microsoft Excel sheet. We were careful to avoid including data from duplicate publications. In the case of suspected data discrepancies, we contacted the relevant corresponding author directly. Data extracted from eligible trials included the following parameters: (1) study characteristics (i.e., first author’s name, year of publication, study location, study design, inclusion and exclusion criteria, and primary findings); (2) participant characteristics in each group (i.e., number of participants, age, sex, comorbidities, and copeptin levels). All detailed information was checked by a third author (L.K.), with disagreements resolved by discussion and consensus.

### 2.4. Quality Assessment

A systematic assessment of bias in the included studies was performed using the Cochrane criteria [16,17]. For this purpose, a tool for Risk Of Bias In Non-randomized Studies—of Interventions (ROBINS-I) [18] was used. ROBINS-I examines seven domains of bias owing to the following: (1) confounding; (2) selection of participants; (3) the classification of interventions; (4) deviations from intended interventions; (5) missing data; (6) measurement of outcomes; and (7) the selection of the reported result. The overall ROBINS-I judgment at domain and study level was attributed to the criteria specified in the ROBVIS tool [19]. The risk of bias (RoB) was performed independently by three reviewers (A.G., L.K., and M.L.); disagreements were resolved by a third reviewer (L.S.) if necessary.

### 2.5. Statistical Analysis

All analyses were performed using Cochranre Review Manager (ver. 5.4, Nordic Cochrane Centre, The Cochrane Collaboration, Copenhagen, Denmark). The Mantel–Haenszel method was used to analyze dichotomous outcomes, and results are reported as odds ratios (ORs) or risk ratios (RRs) with a 95% confidence interval (CI). Continuous outcome differences were analyzed using an inverse variance model with a 95% CI, and values are reported as mean difference (MD). When the continuous outcome was reported in a study as median, range, and interquartile range, we estimated means and standard deviations using the formula described by Hozo et al. [16].

We quantified heterogeneity in each analysis by the tau-squared and I-squared statistics. Heterogeneity was detected with the chi-squared test with n − 1 degree of freedom, which was expressed as I^2^. Values of I^2^ >50% and >75% were considered to indicate moderate and significant heterogeneity among studies, respectively. A random-effects model was used to pool study results independently of the *p*-value for heterogeneity or I^2^ [17]. All *p*-values were two-tailed and considered significant if <0.05.

To evaluate the potential for publication bias, we plotted values against associated standard errors [18] and used Begg’s test to assess the symmetry of the resulting funnel plot [19]. We considered publication bias to be present when the *p*-value was <0.1 in the asymmetry test. However, when a limited number of studies (<10) were included in the analysis, publication bias was not evaluated.

## 3. Results

### 3.1. Study Selection

**Figure 1:**
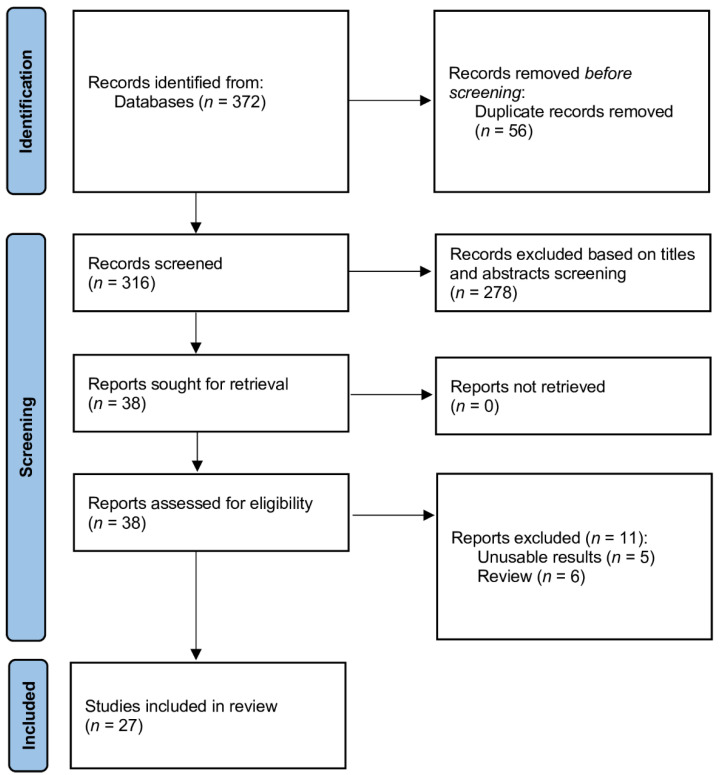
Flow diagram showing stages of the database search and study selection as per Preferred Reporting Items for Systematic Reviews and Meta-Analyses (PRISMA) guidelines.

Figure 1 shows the results of the search and article selection. The final set consisted of 27 eligible studies [20,21,22,23,24,25,26,27,28,29,30,31,32,33,34,35,36,37,38,39,40,41,42,43,44,45,46], including 6 articles conducted in Germany [23,32,34,39,40,42]; 5 articles in France [25,26,31,37,41]; 2 articles in Switzerland [36,45]; 2 articles in Egypt [20,27]; 1 from each of the following countries: Denmark [46], Iceland [44], Korea [33], USA [35], Poland [38], Sweden [43], Spain [21], The Netherlands [29], and Turkey [22]; and 2 international articles [24,28]. The total number of participants was 14,139. All of those studies focused on the copeptin levels in acute coronary syndrome.

Among the included trials, the mean age of patients with and without ACS varied and amounted to 66.1 ± 10.4 versus 62.2 ± 12.7 years, respectively. Males accounted for 71.5% of patients in the ACS group compared with 59.3% in the patients without ACS group. The details of the included studies are summarized in Table 1 and Table S1.

### 3.2. Results of the Meta-Analysis

**Figure 2:**
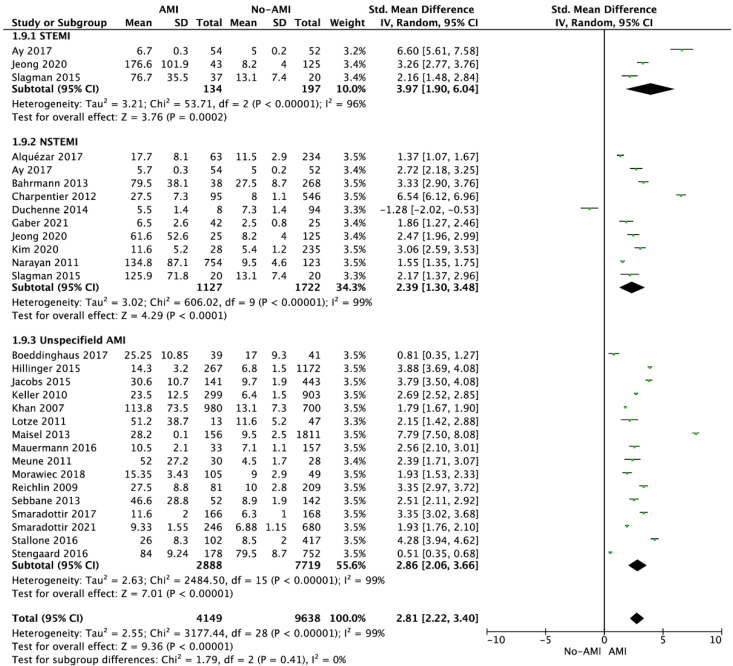
Forest plot of copeptin levels in the AMI and no-AMI groups. The center of each square represents the weighted standard mean difference for individual trials, and the corresponding horizontal line stands for a 95% confidence interval. The diamonds represent pooled results. Legend: CI = confidence interval; SMD = standard mean difference.

The pooled analysis of twenty-six studies showed that copeptin levels in AMI and non-AMI groups varied and amounted to 68.7 ± 74.7 versus 14.8 ± 19.9 pmol/L (SMD = 2.63; 95% CI: 2.02 to 3.24; I^2^ = 99%; *p* < 0.001) [21,22,23,24,25,26,27,28,29,30,31,32,33,34,35,37,38,39,40,41,42,43,44,45,46]. Subanalysis by type of AMI showed that higher concentrations of copeptin in the AMI group versus no-AMIs group were reported across all subgroups: STEMI (82.5 ± 95.9 vs. 7.9 ± 4.5 pmol/L, respectively; SMD = 3.94; 95% CI: 1.87 to 6.01; I^2^ = 96%; *p* < 0.001; Figure 2), NSTEMI (100.8 ± 89.4 vs. 11.1 ± 8.4 pmol/L; SMD = 2.40; 95% CI: 1.31 to 3.49; I^2^ = 99%; *p* < 0.001), as well as in the unspecified AMI group (56.3 ± 62.2 vs. 15.5 ± 21.5 pmol/L; SMD = 2.86; 95% CI: 2.06 to 3.66; I^2^ = 99%; *p* < 0.001).

**Figure 3:**
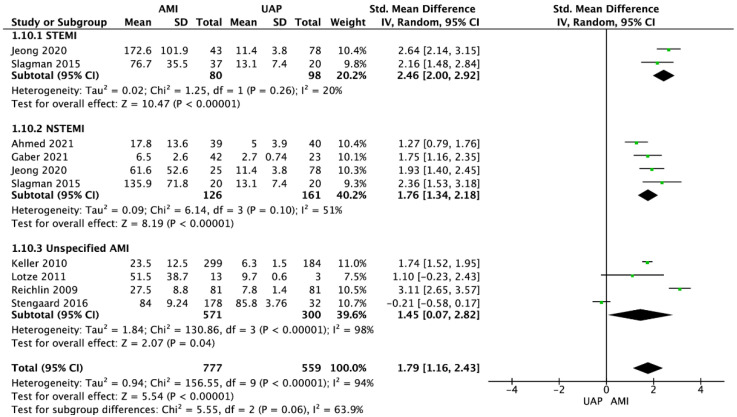
Forest plot of copeptin levels in the AMI and UAP groups. The center of each square represents the weighted standard mean difference for individual trials, and the corresponding horizontal line stands for a 95% confidence interval. The diamonds represent pooled results. Legend: CI = confidence interval; SMD = standard mean difference.

Eight studies reported copeptin levels between AMI and unstable angina pectoris (UAP) groups [20,27,30,31,34,40,42,46]. Pooled analysis showed that mean copeptin levels in the AMI group were higher than in the UAP group 51.9 ± 52.5 pmol/L; 12.8 ± 19.7 pmol/L (SMD = 1.53; 95% CI: 0.86 to 2.20; I^2^ = 95%; *p* < 0.001). Subanalysis showed that copeptin levels were higher in the AMI group compared with the UAP group across all subgroups: STEMI (128.2 ± 91.7 vs. 11.7 ± 4.8 pmol/L respectively; SMD = 2.46; 95% CI: 2.00 to 2.92; *p* < 0.001), NSTEMI (41.5 ± 58.9 vs. 8.8 ± 5.6 pmol/L; SMD = 1.76; 95% CI: 1.34 to 2.18; I^2^ = 51%; *p* < 0.001), and unspecified AMI (43.6 ± 30.1 vs. 15.2 ± 24.5 pmol/L; SMD = 1.45; 95% CI: 0.07 to 2.82; I^2^ = 98%; *p* = 0.04; Figure 3).

**Figure 4:**
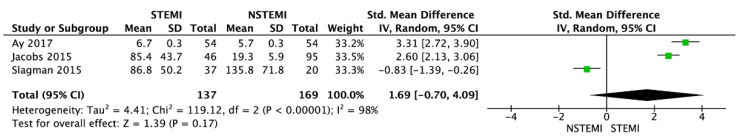
Forest plot of copeptin levels in the STEMI and NSTEMI groups. The center of each square represents the weighted standard mean difference for individual trials, and the corresponding horizontal line stands for a 95% confidence interval. The diamonds represent pooled results. Legend: CI = confidence interval; SMD = standard mean difference.

Three studies [22,29,42] reported copeptin levels in STEMI versus NSTEMI patient groups, which were 54.8 ± 53.0 versus 28.7 ± 46.8 pmol/L (SMD = 1.69; 95% CI: = 0.70 to 4.09; I^2^ = 98%; *p* = 0.17; Figure 4).

## 4. Discussion

According to our study, copeptin assessment is a strong diagnostic tool in patients with AMI thanks to its high negative predictive value (NPV). Ruling out healthy individuals reporting at least chest pain may increase the efficacy of work in the emergency department. However, it was found that a combination of copeptin and hs-cTnT is even more sensitive to rule out NSTEMI, compared with both of those markers tested independently [41]. It was also shown that the double-testing approach has an excellent NPV for short-term risk stratification, and such a strategy is able to improve a triage system in an emergency department. Other authors also showed that copeptin measurement alone exhibits inferior results towards its combination with either N-terminal pro b-type natriuretic peptide (NT-proBNP) or troponin. Although, according to the latest ESC Guidelines, routine measurement of additional biomarkers for diagnostic purposes is not recommended, and assessment of copeptin may add a substantial value to (less sensitive) cardiac troponin [31,47]. Copeptin could be used as a marker to diagnose AMI, but so far, further studies are required to evaluate its potential superiority over the currently available markers of AMI.

In addition to the diagnostic role of copeptin, it acts as a mortality predictor after AMI. In an analysis of 926 patients with AMI, a significant association between the copeptin level and the risk of mortality was found, independently from cortisol and NT-proBNP measurements [44].

Three studies reported a higher level of copeptin in patients with the STEMI compared with the NSTEMI group. That may suggest a positive correlation of copeptin serum concentration with AMI severity. Moreover, the lack of necessity of serial blood sampling is highlighted by some authors [48]. Not only would it reduce the total cost of diagnostic procedures, but also it enables to obviate prolonged monitoring and thus improve the pace of medical care service. Copeptin has been demonstrated to present an acceptable prognostic value for mortality in patients with ACS, but this finding has to be confirmed in a larger multi-marker strategy to evaluate the prognostic value of copeptin for ACS in conjunction and comparison with other well-established biomarkers [44,49].

However, there are some data undermining copeptin utility in the diagnostic path. In some research, it was proved that copeptin measurement substantially does not improve the early diagnosis of AMI and, referring to another study, its accuracy was moderate and inferior to that of hs-cTnT [24,45].

We acknowledge some limitations of our study. Firstly, because of significant heterogeneity of gathered data, it is hard to determine whether the “non-AMI” group was formed by patients suffering from unstable angina, people reporting chest pain at the admission, or healthy individuals. Secondly, the cut-offs for elevated copeptin concentrations differed per study. Therefore, the value of copeptin level in this cohort may be debatable. On this account, it is hard to determine the role of diagnostic and prognostic role of copeptin in AMI. Whereas it seems to be a useful tool, too few data are currently available to classify it as a standard of care, self-sufficient biomarker of AMI. Thereby, further multicenter randomized control trials should be conducted to draw final conclusions.

## 5. Conclusions

Elevated copeptin levels were observed in patients with ACS compared with patients without ACS. Given its clinical value, copeptin levels may be included in the assessment of patients with ACS as well as for the initial differentiation of ACS.

## Figures and Tables

**Table 1:** Characteristics of included studies. Legend: NR = not reported.

Study	Country	Study Design	ACS Group			No-ACS Group		
			No.	Age	Sex, Male	No.	Age	Sex, Male
Ahmed et al., 2021	Egypt	Prospective observational follow-up study	79	58.31 ± 9.61	44 (69.6%)	NR	NR	NR
Alqueézar et al., 2017	Spain	Retrospective observational study	63	72.3 ± 4.3	45 (71.4%)	234	67.3 ± 2.5	154 (88.8%)
Ay et al., 2017	Turkey	Retrospective observational study	108	59.8 ± 10.3	80 (74.1%)	52	55.3 ± 12.6	30 (57.7%)
Bahrmann et al., 2013	Germany	Retrospective observational study	38	82 ± 6	23 (60.5%)	268	80 ± 6	126 (47.1%)
Boeddinghaus et al., 2017	International	Prospective internationalmulticenter study	39	73.5 ± 4.6	31 (79.5%)	41	72.3 ± 6.1	28 (68.3%)
Charpentier et al., 2012	France	Prospectivesingle-center study	95	67 ± 15.6	66 (69.5%)	546	56 ± 16.2	358 (65.6%)
Duchenne et al., 2014	France	Prospective internationalmulticenter cohort study	8	66 ± 16	7 (87.5%)	94	57 ± 55.9	57 (55.9%)
Gaber et al., 2021	Egypt	Prospective case-controlled study	65	61 ± 9.8	35 (53.8%)	25	56.9 ± 15.7	13 (52.0%)
Hillinger et al., 2015	International	Prospective internationalmulticenter study	267	72 ± 3.3	191 (71.5%)	1172	61.3 ± 4.2	795 (67.8%)
Jacobs et al., 2015	The Netherlands	Retrospective observational study	141	65.8 ± 3.5	107 (75.9%)	443	61 ± 3.7	246 (55.5%)
Jeong et al., 2020	Korea	Prospectivesingle-center study	146	62.2 ± 6.5	110 (75.3%)	125	56.7 ± 2.4	77 (61.6%)
Keller et al., 2010	France	Prospectivemulticenter study	483	64.7 ± 10.9	351 (72.7%)	903	59.8 ± 14.3	569 (63.0%)
Khan et al., 2007	Germany	Prospectivesingle-center study	980	62.8 ± 11.8	718 (73.3%)	700	61.9 ± 5.9	409 (58.4%)
Kim et al., 2020	Korea	Prospective observational study	28	67.9 ± 5.1	21 (75.0%)	235	66 ± 3.0	131 (55.7%)
Lotze et al., 2011	Germany	Prospective observational study	13	64.5 ± 11.2	11 (84.6%)	129	71.8 ± 13.6	65 (50.4%)
Maisel et al., 2013	USA	Prospectivemulticenter study	156	61.6 ± 11.4	117 (75.0%)	1811	56 ± 12.8	1001 (55.3%)
Mauermann et al., 2016	Switzerland	Secondary analysis of prospectively collected data	33	74 ± 6	30 (90.9%)	157	72 ± 8	115 (73.2%)
Meune et al., 2011	France	Prospective cohort study	30	61.8 ± 13.6	22 (73.3%)	28	53.7 ± 12.3	15 (53.6%)
Morawiec et al., 2018	Poland	Cohort, cross-sectional study	105	64 ± 2.7	73 (69.5%)	49	62 ± 4.0	27 (55.1%)
Narayan et al., 2011	Germany	Prospective cohort study	754	68.5 ± 10	519 (68.8%)	123	72.3 ± 2.7	82 (66.7%)
Reichlin et al., 2009	Germany	Prospective cohort study	81	68 ± 15	58 (71.8%)	406	61 ± 17	263 (64.8%)
Sebbane et al., 2013	France	Prospective cohort study	52	60.4 ± 7.0	32 (60.8%)	NR	NR	NR
Slagman et al., 2015	Germany	Prospective cohort study	77	56.7 ± 5.8	65 (84.4%)	16	58.5 ± 5.2	11 (68.8%)
Smaradottir et al., 2017	Sweden	Prospective cohort study	166	64 ± 2.3	116 (69.9%)	168	64.5 ± 2.3	115 (68.5%)
Smaradottir et al., 2021	Iceland	Retrospective cohort study	246	77.5 ± 1.3	157 (63.8%)	680	76 ± 1.3	292 (42.9%)
Stallone et al., 2016	Switzerland	Prospective cohort study	102	67.3 ± 3.5	76 (74.5%)	417	57.5 ± 4.0	284 (68.1%)
Stengaard et al., 2016	Denmark	Retrospective study	210	69 ± 2.6	160 (76.2%)	752	65.1 ± 0.4	417 (55.4%)

## Data Availability

Not applicable.

## Supplementary Materials

The following are available online at https://www.mdpi.com/article/10.3390/jcdd9010006/s1; Table S1: Methodology characteristics of included studies; Figure S1: A summary table of review authors’ judgements for each risk of bias item for each study; Figure S2: A plot of the distribution of review authors’ judgements across studies for each risk of bias item.
