# Supplementary material for: Performance of Copeptin for Early Diagnosis of Acute Coronary Syndromes: A Systematic Review and Meta-Analysis of 14,139 Patients
Source: J Cardiovasc Dev Dis. 2021 Dec 27;9(1):6. doi: 10.3390/jcdd9010006 (PMC8780262; doi:10.3390/jcdd9010006)
Supplement: Supplementary file 1 [file jcdd-09-00006-s001.zip › jcdd-1483606-supplementary.pdf]

Copeptin as a biomarker for early diagnosis of acute coronary syndromes: A systematic review and meta-analysis of 14,139 patients

Supplementary Digital File

Content

Table S1. Methodology characteristics of included studies 2

Figure S1. A summary table of review authors' judgements for each risk of bias item for each study. 11

Figure S2. A plot of the distribution of review authors' judgements across studies for each risk of bias item. 12

Table S1. Methodology characteristics of included studies

Study	Inclusion criteria	Exclusion criteria	Primary outcome(s)	Findings
Ahmed et al. 2021	Patients presenting with typical ischemic chest pain within six hours of symptom onset and without ST-segment elevation on ECG.	Patients presenting with ST-elevation myocardial infarction (STEMI) or non-ischemic chest pain and other causes that might influence the serum level of copeptin such as septicemia, end-stage renal disease, hyponatremia (sodium concentration less than 135 mmol/L), ischemic stroke, intracerebral hemorrhage, traumatic brain injury, hemorrhagic shock and vasodilatory shock.	The diagnostic capability of admission copeptin to rule out NSTEMI diagnosis among patients with chest pain.	The combination of copeptin and conventional troponin I aids in early rule out of NSTEMI virtually independent of chest pain onset (CPO) with high NPV in patients presenting within three hours from chest pain onset with excellent prognostic value for risk stratification and prediction of MACEs.
Alquézar et al. 2017	Patients over 18 years of age who consulted for presenting a non-traumatic chest pain, started during the 24 hours prior to the consultation, which was suggestive of myocardial ischemia.	Patients with ST segment elevation or block left bundle branch previously unknown in the electrocardiogram, treatment with thrombolysis, defibrillation or cardioversion prior to arrival at the hospital, kidney failure on dialysis, unstable angina in 2 months prior, coronary artery bypass grafting in the previous 3 months or pregnancy.	Mortality assessed in all included patients for any cause and reinfarction 12 months after the visit to the emergency room by phone call.	The hs-copep assay does not increase the diagnostic or prognostic yield already provided by the hs-cTnt assay in patients suspected of myocardial infarction in the ED.
Ay et al. 2017	Patients aged 18 years or over admitted to the emergency department for AMI.	Patients under the age of 18, patients with chronic renal failure, patients with creatine levels over 1.5 mg/dL,	Differences in serum troponin-I, CK-MB mass, copeptin and CRP levels.	Cardiac troponin remains the gold standard biomarker for the diagnostic evaluation of AMI. Copeptin can be used as a diagnostic marker in patients with

		patients with normal cTnI levels in the follow-up, and patients without AMI.		suspected AMI in combination with other biomarkers, but copeptin alone should not be considered as a single diagnostic marker in patients with suspected AMI.
Bahrmann et al. 2013	All consecutive non-surgical patients aged ≥70 years who were admitted to the emergency department.	Acute STEMI, planned elective coronary revascularization, hospitalization for unstable angina within the preceding 2 months, coronary-artery bypass grafting or percutaneous transluminal angioplasty within the preceding 3 months. Patients were also excluded if they had terminal renal failure requiring dialysis, trauma with suspected myocardial contusion, limited life expectancy < 6 months, or if they did not consent to providing a blood sample for use by the research team.	NS	In unselected older patients presenting to the ED, the additional use of copeptin-us at predefined cut-offs may help to reliably rule out NSTEMI but may not help to increase predicted risk for outcome compared with hs-cTnT alone.
Boeddinghaus et al. 2017	Patients presenting to the ED with symptoms suggestive of AMI (such as acute chest discomfort and angina pectoris) with an onset or peak within the last 12 h and an age >18.	Patients with ST-segment elevation MI, patients with an unknown diagnosis after adjudication (in whom three independent cardiologists were not able to make a definite diagnosis in respect of current guidelines) and at least one elevated hs-cTnT level possibly indicating	2-year all-cause mortality.	About 6–22% of patients presenting with suggestive AMI to the ED have mild hs-cTnT/I elevations at presentation. In contrast to copeptin, the addition of 1h-hs-cTn changes substantially improves the early diagnosis of AMI.

		AMI, as well as patients with missing hs-cTnT or copeptin measurements at 1 h or terminal kidney failure requiring dialysis.		
Charpentier et al. 2012	Patients with suspected ACS presenting at the ED.	Age less than 18 years, ST elevation on a 12-lead ECG, evident traumatic cause of chest pain, skeletal muscle injury within 7 days, previous severe renal impairment, or severe communication problems.	NSTEMI, was diagnosed according to the universal definition of MI by the presence of symptoms of myocardial ischemia and / or relevant ECG changes and an elevated level of cTnI (>0.1 lg / L) on serial testing	Determination of copeptin, in addition to cTnI, improves early diagnostic accuracy of NSTEMI. However, the sensitivity of this combination even using a conventional troponin assay remains insufficient to safely rule out NSTEMI at the time of presentation.
Duchenne et al. 2014	Patients older than 18 years with chest pain suggestive of ACS of <12 h duration since its onset.	Sepsis, shock, lung neoplasms, terminal kidney failure requiring dialysis and hyponatraemia are diseases in which the rate of vasopressin, and thus of copeptin, may be modified.	Copeptin, troponin, myoglobin and creatine kinase values.	In this study, copeptin does not add a diagnostic value at admission to ED for patients with suspected acute coronary syndrome without ST-segment elevation and with hs-cTnT below the 99th centile.
Gaber et al. 2021	Only ACS patients who presented within 4 h of the onset of symptoms.	STEMI patients, ACS patients presenting more than 4 h from pain onset, myocarditis, pulmonary embolism, congestive heart failure, and renal failure.	Circulating copeptin, miRNA-208, and miRNA-499 as possible biomarkers for early detection of unstable angina (UA) and non-ST-segment elevation myocardial infarction (NSTEMI).	Copeptin and miRNA-208 and miRNA-499 expression are promising biomarkers for UA and NSTEMI that present in the first 3 h of pain onset. A combination of these markers with cTn may increase the accuracy of diagnosis by avoiding the gray zone of cTn as a biomarker.
Hillinger et al. 2015	Women and men 18 years old, who presented to the emergency department with symptoms	(a) the final diagnosis remained unclear after adjudication and ≥1 high-sensitivity cardiac troponin T	The incremental value of 1 h copeptin in the rule-out setting (0 h hs-cTnT negative and 0 h copeptin	One-hour copeptin increased neither the safety of the rule-out process nor the NPV in the intermediate-risk setting. In

	suggestive of AMI with an onset or peak within the last 12 h.	(hs-cTnT) concentration was increased, thus possibly indicating the presence of an AMI, or (b) ST-segment elevation elevation myocardial infarction was the adjudicated final diagnosis, as biomarkers are considered of limited clinical value in these patients.	negative) and the intermediate-risk setting (0 h hs-cTnT negative and 0 h copeptin positive).	contrast, the incremental value of 1 h hs-cTnT was substantial in both settings.
Jacobs et al. 2015	All consecutive (>18-year-old) patients presenting to the ED with chest pain within the first 12h after the onset of symptoms.	NS	Cardiac troponin I, copeptin and heart-type fatty acid-binding protein concentrations.	Combining copeptin, heart-type fatty acid-binding protein and cardiac troponin I measurements improves the diagnostic performance in patients presenting with chest pain. Importantly, in patients who present early (<3 h) after chest pain onset, the combination improves the diagnostic performance compared with the standard cardiac troponin I measurement alone.
Jeong et al. 2020	Patients older than 18 years with chest pain onset within six hours of presentation and suspected ACS who presented at the ED.	Patients with traumatic causes of chest pain.	Serum CK-MB, TnI, and copeptin levels.	The combination of TnI and copeptin improves AMI diagnostic performance in patients with early onset chest pain in an ED setting.
Keller et al. 2010	Patients older than 18 years and younger than 85 years of age with angina pectoris or equivalent symptoms.	Trauma or major surgery within the last 4 weeks, pregnancy, intravenous drug abuse, and anemia (hemoglobin <10 g/dl).	Copeptin, troponin T (TnT), myoglobin, and creatine kinase-myocardial band were determined at admission and after 3 and 6 h.	In triage of chest pain patients, determination of copeptin in addition to troponin improves diagnostic performance, especially early after CPO. Combined determination of troponin and copeptin provides a remarkable negative predictive value virtually independent of CPO time; therefore, it aids in early and safe rule-out of myocardial infarction.

Khan et al. 2007	AMI patients admitted to the coronary care unit.	Known malignancy or surgery in the previous month.	The value of both copeptin and NTproBNP for the prediction of death or HF.	The vasopressin system is activated after acute myocardial infarction. Copeptin may predict adverse outcome, especially in those with an elevated NTproBNP (more than 900 pmol/L).
Kim et al. 2020	Patients who presented to the ED with complaints of chest pain were prospectively screened by a single researcher from 8:00 to 17:00 during weekdays. Patients were enrolled in the study if they did not fulfill the following exclusion criteria: age ≤18 years, symptom onset to arrival time ≥12 hours, STEMI on ECG, and refusal to participate.	Not meet inclusion criteria.	copeptin and hs-cTnI assays concentration.	The multi-marker strategy (copeptin and hs-cTnI measurement) was not inferior to serial hs-cTnI measurements in terms of NPV for AMI diagnosis, with a sensitivity and NPV of 100%. Copeptin may help in the early rule-out of AMI in patients with chest pain.
Lotze et al. 2011	Patients with chest pain or other symptoms suggestive of acute MI presenting to the emergency department.	Patients with end-stage renal disease undergoing dialysis.	Troponin T high-sensitive and copeptin levels.	According to this early experience, a single determination of troponin T high-sensitive and copeptin may enable early and accurate exclusion of acute MI in one third of patients, even in an emergency department of a general hospital.
Maisel et al. 2013	Patients who presented with chest pain or ischemic-equivalent symptoms within 6 h of onset of symptoms. Patients >18 years of age were included if the treating physician had suspicion for the diagnosis of ACS.	Symptoms were clearly not related to an ACS (i.e., penetrating chest wounds, crush injury).	Diagnosis of AMI.	Adding copeptin to cTnI allowed safe rule out of AMI with a negative predictive value >99% in patients presenting with suspected acute coronary syndromes. This combination has the potential to rule out AMI in 58% of patients without serial blood draws.

Mauermann et al. 2016	CAD and elective major surgery or ≥2 risk factors for CAD and elective major vascular surgery.	Medication with sulfonylurea derivates or theophylline, current congestive heart failure, current unstable angina pectoris, preoperative hemodynamic instability, emergency surgery, hepatic or renal insufficiency, severe chronic obstructive pulmonary disease, previous enrollment in the study, concurrent enrollment in another randomized con-trolled trial, pregnancy, and absent consent.	The occurrence of myocardial injury, defined as a cardiac troponin T level ≥.03 μg/L on either the first or second postoperative day without evidence of an alternative explanation of troponin elevation.	Copeptin (≥9.6 pmol/L) was associated with significantly higher rates of myocardial injury and improved risk stratification in patients scheduled for non-cardiac surgery with non-elevated preoperative troponin.
Meune et al. 2011	Consecutive patients who were admitted to the Department of Cardiology for suspected recent ACS, defined as chest pain of ≤ 6 hours' duration since onset, suggestive of myocardial ischaemia, and lasting > 5 minutes at rest or upon minimal exertion.	Patients presenting after a cardiac arrest or with ST-segment elevation myocardial infarction.	Copeptin and hs-cTnI assays concentration.	This prospective study demonstrated that a dual marker strategy that combines hs-cTnT with copeptin increased slightly the detection of acute coronary syndrome at admission.
Morawiec et al. 2018	Chest pain of a minimum of 5-minute duration with beginning during the last 6 hours.	The presence of persistent ST-segment elevation in an electrocardiogram at admission or major conditions with proved influence on copeptin elevation (e.g., end-stage renal disease, sepsis, anaemia, and hyponatremia).	The diagnosis of AMI.	The combination of baseline hs-TnT, copeptin, and the mHS has an excellent sensitivity and NPV for short-term risk stratification. Such approach might improve the triage system in emergency departments and be a bridge for inclusion to serial blood sampling algorithms.

Narayan et al. 2011	(a) NSTEMI patients who were included in the original LAMP study, which included both NSTEMI and STEMI patients, who were recruited between March 2000 and July 2005; and (b) a new group of NSTEMI patients who were recruited between August 2005 and April 2007. As a control group, plasma copeptin and NT-proBNP were also measured in 82 healthy males over 65 and 41 females over 70 years of age.	Patients with known malignancy or surgery in the previous month.	The utility of plasma copeptin and NT- proBNP level for prediction of the end point of all- cause mortality at 6 months.	Plasma copeptin is a novel independent prognostic biomarker in patients following non-ST elevation acute coronary syndrome and improves the early prognostic accuracy of the GRACE score compared with NT- proBNP.
Reichlin et al. 2009	Patients presenting to the ED with symptoms suggestive of AMI such as chest pain and angina pectoris with onset or peak within the last 12 h.	Patients with terminal kidney failure requiring dialysis.	Copeptin, the C-terminal part of the vasopressin Prohormone levels.	The additional use of copeptin seems to allow a rapid and reliable rule out of AMI already at presentation and may thereby obviate the need for prolonged monitoring and serial blood sampling in the majority of patients.
Sebbane et al. 2013	ED patients with acute chest pain enrolled in an ongoing plasma bank of patients with chest pain, as described earlier. Adult patients with chest pain and onset within 12 hours of presentation to the ED or to management by the French prehospital emergency service.	Patients with traumatic causes of chest pain.	Diagnostic performances of us-copeptin combined with hs-cTnT were assessed using logistic regression.	Assessment of us-copeptin combined with hs-cTnT on ED admission could allow safe and early rule out of NSTEMI for patients with negative results on both markers and help identify patients who may be suitable for discharge.
Slagman et al. 2015	Patients who experienced acute chest pain and had a clinically high suspicion of AMI.	Patients with anemia (hemoglobin <10 g/dL) or known chronic renal failure,	Copeptin and high-sensitivity cardiac troponin T (hs-cTnT) levels.	Our analysis is the first to show a consistent early increase in copeptin at first medical contact in the ambulance and a decrease to routine values within 12–36

		age >80 years, or life expectancy <6 months.		h in patients presenting early with spontaneous AMI.
Smaradottir et al. 2017	Patients admitted to the coronary care units for suspected acute myocardial infarction.	Individuals admitted during weekends and holiday seasons, and excluded all individuals who had known diabetes mellitus or who lived outside the catchment area, or who had serum creatinine concentrations of 200 qmol/L or more, or were older than age 80 years.	(1) total mortality; (2) cardiovascular mortality defined as death from myocardial infarction (MI), stroke, aortic dissection or sudden death without any obvious reason; and (3) major cardiovascular. event defined as the first occurrence of AMI, stroke, severe congestive heart failure or cardiovascular death	Copeptin levels are highest among acute myocardial infarction patients with glucose disturbances and predict an adverse prognosis in unadjusted analyses. These findings imply that raised copeptin reflects stress rather than acting as a pathogenic factor for glucose abnormalities.
Smaradottir et al. 2021	Patients with MI.	Blood samples for copeptin analysis were unavailable.	Cardiovascular events, and secondary endpoints were total mortality, heart failure and MI.	Copeptin was higher in subjects with previous MI regardless whether previously recognized or not. Copeptin correlated weakly with cortisol and NTproBNP, and was independently associated with total mortality. This indicates that the prognostic implications of copeptin are not only mediated by heart failure or stress, supporting the assumption that copeptin is a marker of general vulnerability.
Stallone et al. 2016	Patients older than 18 years of age presenting to the ED with symptoms suggestive of AMI	Patients with end-stage renal failure were excluded. If the final diagnosis remained unclear after adjudication	Hs-cTnT and copeptin levels.	The additional use of copeptin on top of hs-cTnT seems to lead to a small increase in NPV, but no increase in AUC. Routine

	with an onset or peak within the last 12 hours.	and at least one level of high-sensitivity cardiac troponin T (hs-cTnT) exceeded 14 ng/L during serial sampling.		use of copeptin in early presenters does not seem warranted.
Stengaard et al. 2016	Ongoing or prolonged periods of chest discomfort within the past 12 h, acute dyspnea in the absence of known pulmonary disease, or clinical suspicion of AMI.	NS	Hs-cTnT and copeptin levels.	A future application of hs-cTnT and copeptin measurement, performed already in the prehospital phase, could potentially improve the prehospital diagnostic and prognostic classification of patients with a suspected AMI

Legend: ACS = acute coronary syndrome; AMI = acute myocardial infarction; CAD = coronary artery disease; CPO = chest pain onset; ECG = electrocardiogram; ED = Emergency Department; MI = myocardial infarction; NS = not specified;

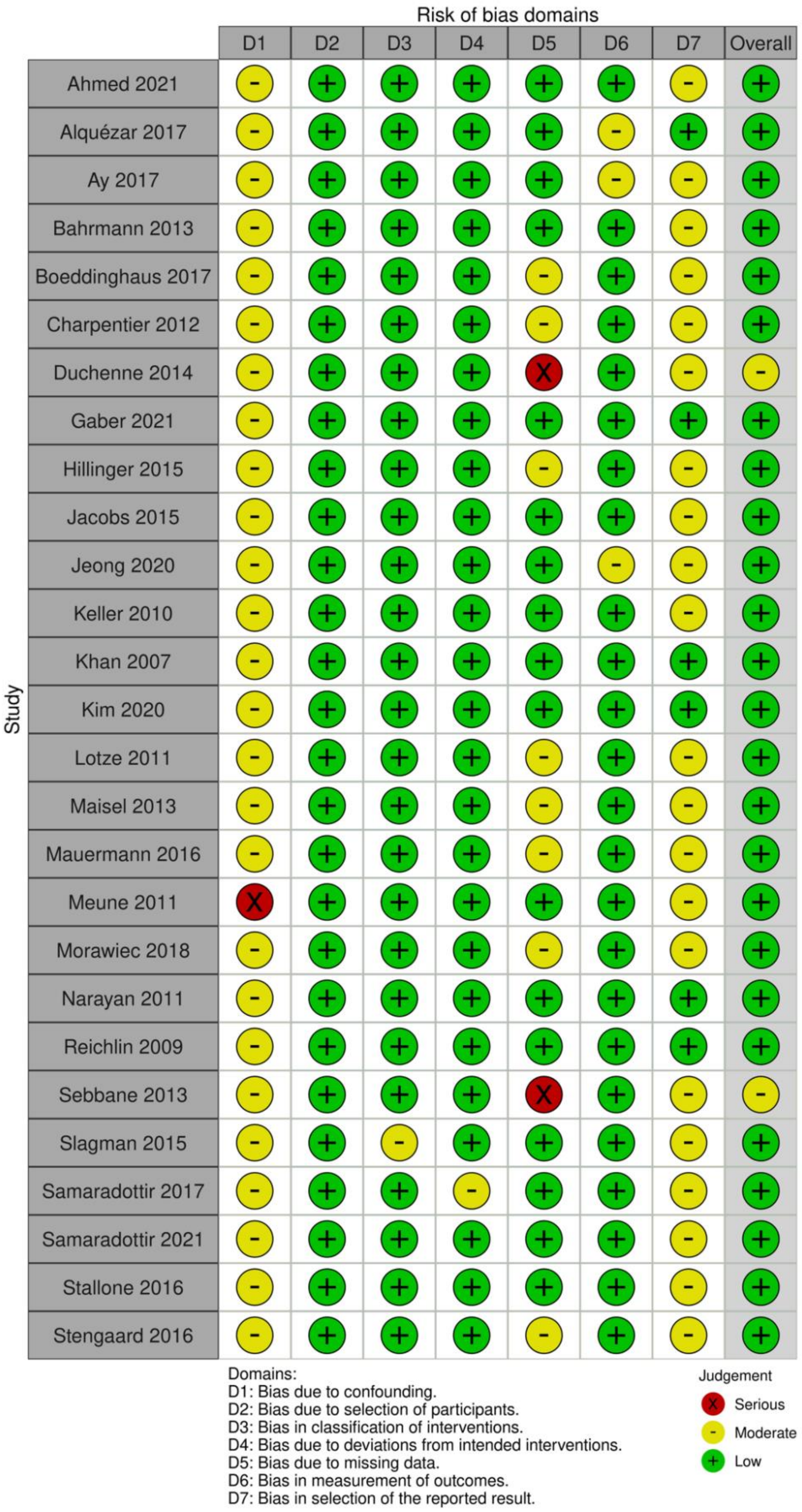

Figure S1. A summary table of review authors' judgements for each risk of bias item for each study.

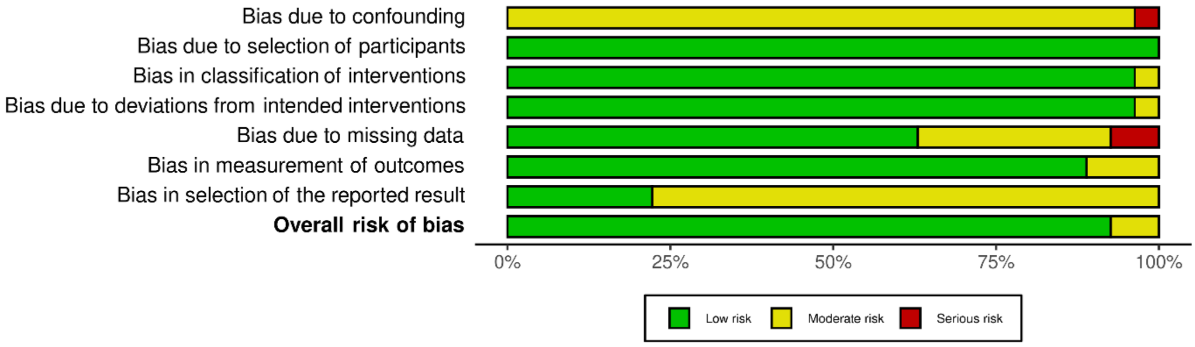

Figure S2. A plot of the distribution of review authors' judgements across studies for each risk of bias item.
